# Motion‐compensated diffusion encoding gradients for segmented thick‐slab 3D magnetic resonance diffusion‐weighted imaging of the brain

**DOI:** 10.1002/mp.70367

**Published:** 2026-03-03

**Authors:** Jens Johansson, Kerstin Lagerstrand, Hanna Hebelka, Stephan E. Maier

**Affiliations:** ^1^ Department of Radiology Institute of Clinical Sciences Sahlgrenska Academy University of Gothenburg Gothenburg Sweden; ^2^ Department of Medical Physics and Biomedical Engineering Sahlgrenska University Hospital Gothenburg Sweden; ^3^ Department of Medical Radiation Sciences Institute of Clinical Sciences Sahlgrenska Academy University of Gothenburg Gothenburg Sweden; ^4^ Department of Radiology Sahlgrenska University Hospital Gothenburg Sweden; ^5^ Department of Radiology Brigham and Women's Hospital Harvard Medical School Boston Massachusetts USA

**Keywords:** acceleration, gradient moment, moment nulling, segmented 3D‐DWI, velocity

## Abstract

**Background:**

Routine clinical magnetic resonance diffusion‐weighted imaging (DWI) is generally performed with 2D echo planar sequences. A single thick‐slab 3D approach could offer higher signal‐to‐noise ratio and better slice resolution. This has not been adopted due to the difficulty to avoid motion‐induced phase error that interfere with multi‐shot spatial encoding.

**Purpose:**

To introduce a new approach for 3D brain DWI: rather than relying on navigator echoes for phase correction, moment‐nulled diffusion encoding gradients are used to minimize phase variations at the source.

**Methods:**

A standard 2D echo planar imaging sequence was modified to incorporate moment‐nulled diffusion encoding gradients and a second phase encoding gradient for spatial multi‐shot encoding along the slice select direction. The single thick‐slab 3D diffusion‐weighted imaging sequence was evaluated with brain scans in healthy volunteers on a 3 Tesla scanner.

**Results:**

Incorporation of both first and second order moment nulling achieved substantial, albeit not comprehensive, reduction of motion‐related ghosting artifacts. Without such motion compensation or with first order moment nulling only, motion‐related artifacts were consistently more severe. Even though the approach comes with a penalty in echo time—at a diffusion weighting of 1000 s/mm^2^, 119 ms for the moment‐nulled 3D acquisition versus 82 ms for the conventional 2D acquisition—the measured ratio between SNR_3D_ and SNR_2D_ for a 92‐slice scan was 0.99.

**Conclusions:**

This proof‐of‐concept shows that first and second order moment nulling may be a viable avenue for enabling 3D diffusion imaging. At higher slice numbers the SNR_3D_ is expected to clearly surpass corresponding SNR_2D_. However, further investigation into echo time reduction and correction of residual phase variations is needed before the 3D approach is viable for translation into a clinical setting. Specifically, with higher gradient strength shorter echo times can be realized. Moreover, this reduces third and higher order gradient moments and associated residual phase shifts.

## 1 INTRODUCTION

Diffusion‐weighted imaging (DWI) predominantly relies on a single‐shot 2D echo‐planar imaging (EPI) data acquisition scheme. The attainable spatial resolution of single‐shot 2D DWI falls short off what can be achieved with other MR imaging contrasts, such as T_1_ and T_2_‐weighted imaging. A 3D diffusion acquisition would allow for superior signal‐to‐noise ratio (SNR), higher spatial resolution, and isotropic voxel dimensions^1^, ^2^, ^3^, ^4^, ^5^ for improved reformatting along arbitrary planes. Higher detail and, consequentially, less partial volume effects are beneficial for anatomical rendering and conspicuity of small lesions.

However, a high‐resolution 3D acquisition cannot be performed in a single readout and, therefore, requires segmentation. With a multi‐shot readout approach, the shot‐to‐shot phase variations that arise from bulk tissue movement^6^ in the presence of standard motion probing diffusion gradients invariably interfere with spatial encoding and lead to pronounced ghosting artifacts. One approach to overcome this limitation is to perform the 3D acquisition for a thin slab.^1^, ^7^ The thickness is limited by the assumption that for thin slabs motion‐related through‐slice phase variation is minimal and that hence in‐plane 2D phase navigator correction suffices.^1^ Another shortcoming of the thin‐slab approach is that the merging of separate slabs introduces slab boundary artifacts.^2^, ^8^, ^9^ Cardiac gating has been proposed to enable thicker slabs.^10^ A 3D navigator is not practical since the inherent low sampling bandwidth of a third sampling direction results in considerable distortions and/or inadequate k‐space coverage.^11^

An entirely different approach is to repetitively employ a diffusion preparation sequence to generate diffusion‐weighted longitudinal magnetization followed by an independent excitation and segmented readout, which has been demonstrated both in 2D^12^ and 3D.^13^ This preparation approach sacrifices half the signal or requires repeat measurement with phase cycling.^13^ Balanced steady state free precession has also been proposed for 3D segmented diffusion imaging, but is constrained by the limited diffusion weighting that can be attained.^14^

We propose an alternative approach based on motion‐compensated diffusion encoding gradients. The goal is to minimize motion‐related shot‐to‐shot phase variations at the source with the compensation of motion effects through moment‐nulling of the diffusion encoding gradients^15^ so that navigation can be simplified or completely omitted. In the early phase of development of robust diffusion imaging sequences, a 2D‐segmented approach for brain imaging with velocity compensation using bipolar diffusion gradients has been reported^16^, ^17^ and more recently it has been applied in the liver.^18^ Time‐efficient velocity and acceleration‐compensated diffusion encoding based on uneven lobe duration bipolar waveforms with low sensitivity for higher order motion terms has been introduced for single‐shot diffusion imaging in the heart.^19^ This usage focused on the elimination of phase dispersion related signal‐loss caused by non‐uniform tissue motion. Recently multi‐shot 2D imaging in brain with such bipolar waveforms at high gradient amplitude has been reported.^20^ Here we report initial experience with a single thick‐slab segmented 3D‐DWI whole‐brain acquisition that uses motion‐compensated diffusion encoding rather than navigator echoes for the reduction of phase errors.

## 2 THEORY

**FIGURE 1:**
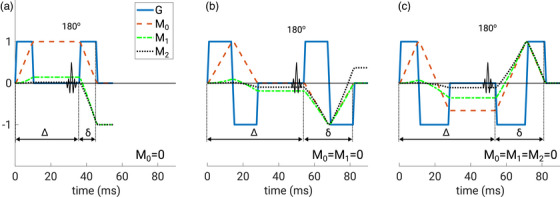
Diffusion encoding schemes are shown for zeroth (a), zeroth and first (b), and zeroth, first and second (c) order moment‐nulling. The time course reflects the actual timings that were realized with 69 mT/m gradient amplitude, a b‐value of 1000 s/mm^2^, and an imaging matrix of 144×144 with full echo sampling. Red lines indicate the time course of the relative zeroth order moment M_0_, green lines the time course of the relative first moment *M*_1_, and black lines the time course of the relative second moment *M*_2_. Only in case (c) are all of these moments compensated for, that is, each moment equals zero at the end of diffusion encoding. The realized gradient duration δ and hence echo time, in (b) and (c) is almost equal. In (c) a remarkably short δ results with the use of the dead‐time prior to the 180° pulse. This maximizes the gradient separation ∆ and enables a shorter δ and echo time. Meanwhile, in case (b) changing ∆ has no such effect.

Diffusion encoding gradients render a sequence sensitive for displacement on a molecular level, which permits the measurement of diffusivity. Invariably, the same diffusion encoding gradients also sensitize a sequence for bulk motion. The resulting signal phase accumulation in a diffusion encoding experiment can be expressed as:

$$\varphi \;\left(\vec{r}\left(t\right)\right)=\gamma \int_{0}^{T}\vec{G}\left(t\right)\vec{r}\left(t\right)dt$$

where $\vec{r}\left(t\right)$ represents the time‐dependent spin position, *γ* the gyromagnetic ratio, *T* the total duration of the diffusion encoding gradient, and $\vec{G}\left(t\right)$ the time‐dependent gradient trajectory. This phase shift can lead to a signal loss if it is spatially non‐uniform and to ghosting artifacts if it varies during a multi‐shot acquisition. To better understand the phase shifts that result with various gradient configurations, it is helpful to decompose the motion into its derivatives using a Taylor expansion of $\vec{r}\left(t\right)$. The observed phase shift can thus be rewritten as:

$$\varphi \;\left(\vec{r}\left(t\right)\right)=\gamma \sum_{n=0}^{\infty }\frac{{\vec{r}}^{\left(n\right)}}{n!}M_{n}$$

and where

$$\;{\vec{M}}_{n}=\int_{0}^{T}\vec{G}\left(t\right){\left(t\right)}^{n}dt$$

signifies the gradient moment of order *n*. In order to avoid a signal loss of stationary tissue, diffusion encoding gradients need to be zeroth‐order compensated, that is, $M_{0}=0$. The simplest configuration of such a gradient is a monopolar gradient, where the gradient is switched on with equal amplitude and for equal duration on each side of the inverting radio‐frequency pulse (see Figure 1a). This configuration, while time‐efficient and thus useful in attaining a short echo time, is prone to introduce significant phase shifts from velocity and higher order motion derivatives. At the expense of gradient duration, the nulling of additional moments higher than *M_0_* can be attained with additional gradient lobes.^21^, ^22^, ^23^ A bipolar velocity‐compensated ($M_{1}=0$) diffusion encoding gradient with equal duration for all lobes is presented in Figure 1b and a very time‐efficient bipolar configuration with uneven lobe duration that is compensated for both velocity and acceleration ($M_{1}=M_{2}=0)$ in Figure 1c.

Diffusion encoding is determined by the b‐value, which in turn depends on the gradient amplitude and the gradient lobe duration. Generally, for a given b value, an increase in gradient amplitude prompts a shorter duration of the gradient lobes. For the present application it is important to understand how this affects the gradient moments and the associated phase shifts in the presence of motion. Since analytic expressions for b‐value and moment integrals of diffusion encoding gradients tend to be complicated, particularly if gradient rise times are considered, it will in the following be helpful to resort to a very basic, hypothetical gradient configuration, that is, a no‐gap monopolar gradient composed of two adjacent rectangular gradient pulses of duration *δ*. In this case, the b‐value integral follows the simple expression $b=2\gamma^{2}G^{2}\delta^{3}/3$. Consequentially, to maintain a given b‐value, the scaling of the gradient amplitude by scaling factor *s* requires reciprocal scaling of the timing parameter *δ* by ${\left(1/s\right)}^{2/3}$. It follows readily that the gradient moment *M*_n_ then scales with the factor ${\left(1/s\right)}^{(2n-1)/3}$. It is thus clear that for a constant b‐value, an increase in gradient amplitude and commensurate reduction in gradient duration leads to a reduced gradient moment, which is favorable if minimization of the motion‐related phase shifts is the goal. Moreover, it can be recognized that this gradient moment scaling factor decreases with order *n* according to a power law. In other words, higher order motion‐related phase shifts decrease over‐proportionally as for a given b‐value the gradient amplitude is increased to attain shorter gradient duration.

**FIGURE 2:**
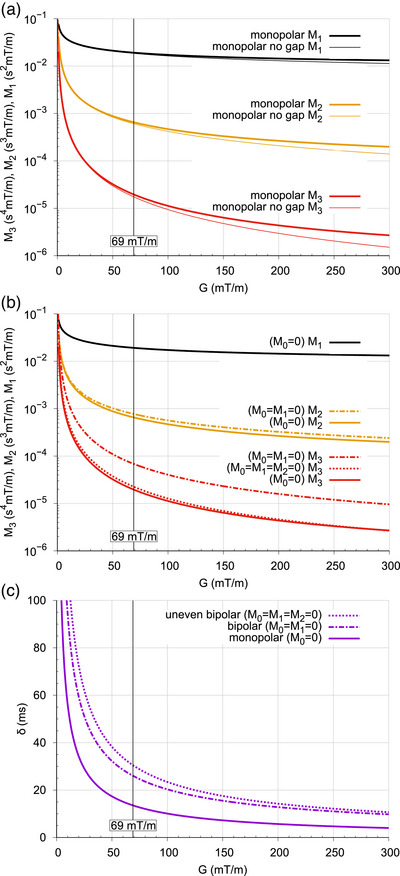
Comparison of moments M_1_, M_2_, and M_3_ (a and b) and timing δ (c) as a function of gradient amplitudes up to 300 mT/m assuming a b‐value of *b* = 1000 s/mm^2^ and rectangular‐shaped gradient lobes. The gradient amplitude employed for the present work is indicated with a vertical line. In (a) the configuration termed “monopolar no gap” is the analytically easily solvable reference scenario that is compared with a conventional monopolar pulse using a time gap of 7 ms to accommodate a radio‐frequency inversion pulse. In (b) the moments of a conventional monopolar gradient configuration are compared with a bipolar (*M*_0 _= *M*_1 _= 0) and an uneven bipolar (*M*_0 _= *M*_1 _= *M*_2 _= 0) gradient configuration and in (c) the resulting δ time interval is shown. A time gap of 7 ms to accommodate a radio‐frequency inversion pulse was assumed for all configurations in Figure 1(b) and (c). The reduction of the moments *M*_1_, *M*_2_, and *M*_3_ and the δ time interval with increasing gradient amplitude is particularly apparent in the lower gradient amplitude range. Since this descent with gradient amplitude becomes steeper with growing order of the moment, an expanding spread of the moment values is evident for increasing gradient amplitudes. When compared to the standard monopolar configuration, the use of an even bipolar gradient trajectory for velocity compensation results in a minor increase of the *M*_2_ moment and a many‐fold increase of the *M*_3_ moment. Meanwhile, for the uneven bipolar configuration the *M*_2_ moment is nulled and the *M*_3_ moment is comparable to the *M*_3_ moment of the monopolar configuration.

To illustrate this behavior, gradient timing *δ* and gradient moments *M*_1_, *M*_2_, and *M*_3_ were computed for different gradient configurations with a b‐value of 1000 s/mm^2^ (see Figure 2). This computation was performed for a wide range of gradient amplitudes up to 300 s/mm^2^.

## 3 METHODS

### 3.1 Pulse sequence design

**FIGURE 3:**
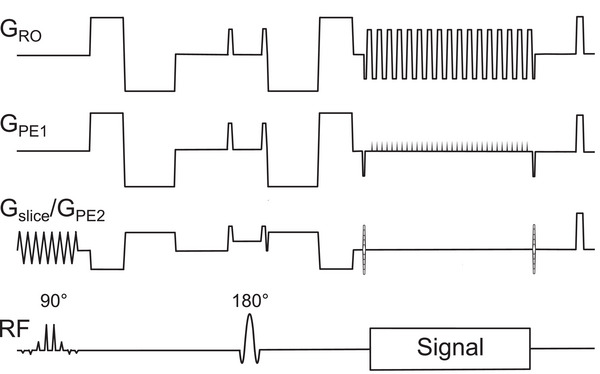
The 3D segmented EPI DWI pulse sequence diagram with first and second order moment‐nulled diffusion encoding gradients. Gradient encoding axes along read‐out (RO), first phase encoding (PE1), and second phase encoding (PE2) direction are depicted together with the radiofrequency (RF) axis.

The 3D multi‐shot sequence shown in Figure 3 was developed based on a 2D product diffusion imaging sequence with single‐shot echo‐planar readout. The modifications included the addition of a second phase encoding gradient for spatial multi‐shot encoding along the slice select direction and the optional replacement of the conventional monopolar diffusion‐encoding gradients with moment‐nulled diffusion‐encoding gradients (Figure 1). Analytic solutions with consideration of ramp times were formulated for the b‐value and gradient moments of an even bipolar ($M_{1}=0$) and an uneven bipolar gradient configuration ($M_{1}=M_{2}=0$). For the uneven bipolar configuration, the incorporation of gradient ramp times requires an iterative solver to compute the plateau times for a given b‐value. This was accomplished with a Levenberg–Marquart algorithm, implemented directly in the pulse sequence using the C/C++ Minpack library.^24^ The first part of the diffusion gradient was applied right after the completion of the spectral‐spatial slice selection pulse and by employing an optimized delay between this gradient and the refocusing pulse the shortest possible echo time could be realized.

### 3.2 Diffusion‐weighted MR brain scans

The study was approved by the Swedish ethical review board (Dnr 2020‐00029) and written informed consent was obtained from each participant. Diffusion‐weighted data without cardiac gating was collected with the 3D multi‐shot EPI‐DWI sequence on a 3 Tesla scanner (Premier, GE Healthcare, Milwaukee WI, USA) with a nominal maximum gradient amplitude of 80 mT/m and a maximum slew rate of 200 mT/m/ms. A total of six brain exams were performed. Four healthy volunteers aged between 30 and 39 years participated in the study, that is, one volunteer underwent three exams. In each volunteer the three different diffusion gradient configurations, that is, $M_{0}=0$, $M_{0}=M_{1}=0,$ and $M_{0}=M_{1}=M_{2}=0$, respectively, were evaluated at an actual maximum amplitude of 69 mT/m. The acquisition was performed with a slab thickness of 120 mm at a FOV of 22×22×14 cm^3^ and a matrix size of 144×144×92 (1.5 mm isotropic resolution, 92 shots). With a TR of 520 ms the scan time amounted to 3:12 min. To evaluate the effect of TR an additional scan for $M_{0}=M_{1}=M_{2}=0$ was performed in one subject with a TR of 1000 ms and a scan time of 6:09 min. Moreover, in one subject a higher resolution scan with the configuration $M_{0}=M_{1}=M_{2}=0$ was obtained using a slab thickness of 100 mm, a FOV of 24×24×120 cm^3,^ and a matrix size of 196×196×150 (1.2×1.2×0.8 mm^3^ resolution, 150 shots). The high‐resolution scan was performed with a TR of 1000 ms and a scan time of 10:01 min. All scans employed a 48‐channel head coil with two‐fold parallel coil acceleration along *k_y_*. Diffusion encoding was performed with a b‐value of 1000 s/mm^2^ for each of three orthogonal diffusion encoding directions, whereas a b‐value of 0 s/mm^2^ was measured only once. In one subject the scan with 1.5 mm isotropic resolution was repeated with different settings of the maximum b‐value, that is, 64, 160, 400, and 1000 s/mm^2^, which resulted in that the respective *M*_3_ moment was 0.26, 0.60, 1.46, and 3.78∙10^−5^ s^4^mT/m. With every scan the full echo was sampled and depending on gradient configuration the resulting echo times for the scans with *b* = 1000 s/mm^2^ were 83 ms for $M_{0}=0$, 119 ms for $M_{0}=M_{1}=0,$ and 119 ms for $M_{0}=M_{1}=M_{2}=0$, respectively. The high‐resolution scan required a TE of 155 ms. Finally, with each exam, a conventional single‐shot 2D‐DWI data set (TE 82 ms) at 1.5 mm isotropic resolution with matching coverage was acquired for comparison. This was done at an equivalent scan time of 3:12 min using a TR of 11451 ms, one acquisition without averaging for *b* = 0 s/mm^2^ and five averaged acquisitions for each diffusion encoding direction with *b* = 1000 s/mm^2^.

Five 3D‐DWI scans were performed sequentially in one subject to assess intra‐subject repeatability for each motion compensation configuration. Three sequential 2D single‐shot DWI scans of the same subject served as reference data. Signal consistency across repeated scans was evaluated by pixel‐wise comparison of mean pixel brain signal and the root mean‐squared error. This data was also used to assess the artifact reduction, for different moment nulling approaches, with a one‐way ANOVA test followed by the post‐hoc Fisher's LSD (*p* < 0.05).

For SNR evaluation, 2D and 3D scans were repeated with all RF pulses disabled to obtain noise maps. The Rayleigh‐corrected mean within the area representative for the brain and the mean brain signal from corresponding regular slices was used to compute SNR. Theoretical SNR values were derived using T1 = 1100 ms,^25^ T2 = 75 ms^26^ and analytical equations from Engström et al.^2^

Image raw data obtained with the segmented 3D sequence were reconstructed offline with in‐house developed programs using MatLab (2023b, MathWorks, Natick, MA, USA) in combination with the Orchestra reconstruction programming suite (GE healthcare, Waukesha, WI) and the Berkeley Advanced Reconstruction Toolbox (BART version 0.8.00).^27^ After regular EPI data processing such as row flipping, re‐gridding of data and Nyquist correction the 3D k‐space data was directly, without further correction, Fourier‐transformed into the image domain. Each aliased 2D slice was then unaliased by parallel imaging reconstruction using the BART function pics.

## 4 RESULTS

**FIGURE 4:**
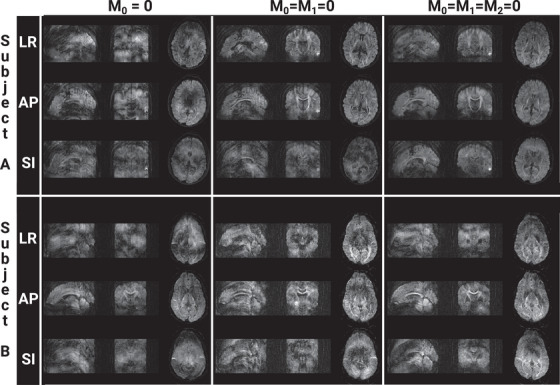
Diffusion‐weighted images (*b* = 1000 s/mm^2^) of two subjects obtained with the segmented 3D diffusion scan using different gradient waveforms for diffusion encoding. Images have been reformatted for the sagittal, coronal, and axial plane, respectively, and are presented for diffusion encoding directions (LR = left‐right, AP = anterior‐posterior, and SI = superior‐inferior) with no (left), first (middle), and first and second (right) order moment nulling. Although images obtained with first and second order moment nulling are not artifact‐free, a clear reduction of artifacts can be appreciated compared to images obtained with no motion compensation or first order moment nulling only. The resolution is 1.5 mm isotropic.

**FIGURE 5:**
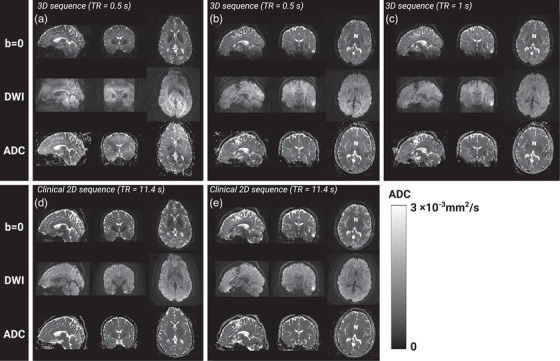
Example of T2‐weighted images (*b* = 0), trace diffusion‐weighted images, and ADC maps of the sagittal, coronal, and axial plane generated from the same segmented 3D scans with first and second order moment nulling as shown in Figure 4 for Subject A (a) and Subject B (b). Images in (c) resulted from such 3D scan with a TR of 1000 ms in Subject B. For comparison, the corresponding reformatted image data from the conventional 2D axial scan is shown for Subject A in (d) and for Subject B in (e).

**FIGURE 6:**
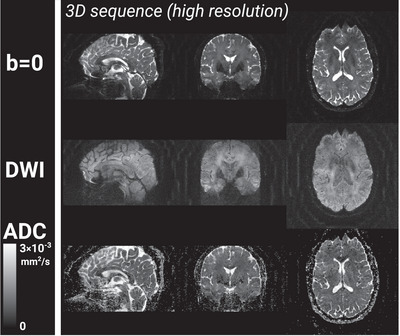
Example of T2‐weighted (b0) images, trace diffusion‐weighted images, and ADC maps of the sagittal, coronal, and axial plane originating from the 1.2 × 1.2 × 0.8 mm^3^ high‐resolution 3D scan in Subject C.

**FIGURE 7:**
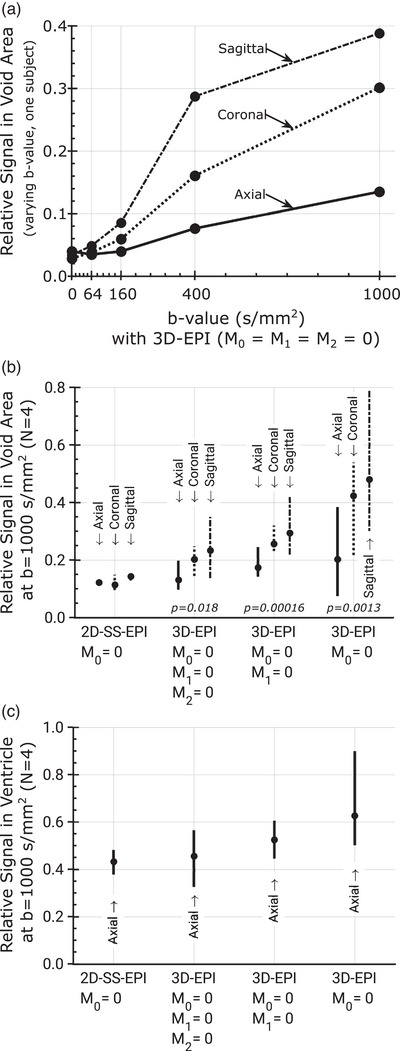
Ghosting artifacts in signal void areas (a, b) and ventricle(c) areas on trace diffusion‐weighted images compared to the average signal in the brain. Signal outside the brain was measured on axial, coronal, and sagittal views, within a narrow rectangular ROI along the FOV edge and spanning over five off‐center adjacent slices, orthogonal to the first phase encode direction (axial view) or the second phase encode direction (coronal and sagittal view). This void area is expected to exhibit no signal, except the signal that stems from ghosting artifacts. The measured signal was set in relation to the average brain signal in five central adjacent slices of the respective view. Graph (a) shows the results of multiple b‐value experiments in one subject and (b) the mean and range (min, max) of the relative signal in four subjects at *b* = 1000 s/mm^2^. For comparison, the relative void signal in the *b* = 0 s/mm^2^ images was roughly half of that observed in the 2D‐SS‐EPI *b* = 1000 s/mm^2^ trace diffusion‐weighted images. The p‐values refer to the comparison of the relative signal in the void areas (axial, coronal, and sagittal combined) of the 3D approaches with the respective values of the established 2D approach. Graph (c) shows for the same four subjects the mean and range (min, max) of the signal in the ventricles relative to the average brain signal measured in one axial slice at *b* = 1000 s/mm^2^.

Images with varying degree of image quality were reconstructed from all segmented 3D diffusion scans. Sagittally, coronally, and axially reformatted examples of diffusion‐weighted images for each diffusion encoding direction measured in two subjects with the three different gradient configuration schemes, that is, $M_{0}=0$, $M_{0}=M_{1}=0,$ and $M_{0}=M_{1}=M_{2}=0$, respectively, are shown in Figure 4. Ghosting artifacts are most evident in the sagittal and coronal plane. Corresponding *b *= 0, trace DWI, and ADC maps from the same two subjects are presented in Figure 5 along with the matching axial images of the 2D scan. Image contrast differences due to TR change are also documented in Figure 6 for *b* = 0 and the trace diffusion‐weighted images. Images of the high‐resolution scan are shown in Figure 6. Figure 7 provides an overview about the observed ghosting artifact intensity for different scan scenarios and across subjects. Phantom scans showed no obvious artifacts related to motion (data not shown).

**FIGURE 8:**
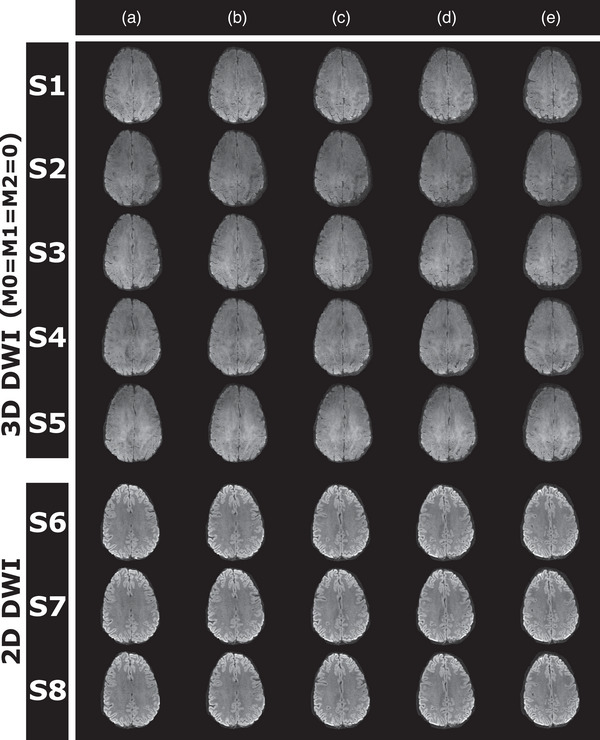
Example trace diffusion‐weighted slices (a–e) of five consecutive 3D‐DWI scans (S1–S5) and three consecutive 2D‐DWI scans (S6–S8) performed in one subject.

With applied apodization filter, trace *b* = 1000 s/mm^2^ images of repeated scans (Figure 8) show a signal variation of approximately 2.4% for 2D‐DWI, corresponding to a Signal‐to‐Artifact and Noise Ratio (SA&NR) of 42 (1/0.024). In comparison, 3D‐DWI with moment nulling up to the second order exhibited a lower overall SA&NR of 7.7, which decreased to 4.9 with moment nulling up to the first order, and 5.4 with moment nulling for zeroth order only. The SA&NR values for individual encoding directions are provided in Table 1. With moment nulling up to the second order (*M*_0 _= *M*_1 _= *M*_2 _= 0) compared to zeroth‐order nulling only (*M*_0 _= 0) artifacts were significantly reduced. Compared to moment nulling up to the first order (*M*_0 _= *M*_1 _= 0) artifacts were also significantly reduced, except for diffusion encoding along the frequency encode direction, where a small increase of artifacts resulted. Notably, the improvement with nulling up to the first order (*M*_0 _= *M*_1 _= 0) compared to zeroth‐order nulling only (*M*_0 _= 0) was inconsistent. In particular, artifacts for the combined images and along all directions except for the frequency encode direction were worse. Noise alone without applying an apodization filter would have resulted in 3.6% signal variation for 2D‐DWI and 3.7% for 3D‐DWI (see Table 2). The measured 3D‐to‐2D SNR ratios were 1.06 (*b* = 0 s/mm^2^) and 0.99 (*b* = 1000 s/mm^2^), aligning with theoretical values of 0.98 and 0.86, respectively (see Table 2).

**TABLE 1:** Signal to artifact and noise ratios for each individual diffusion encoding directions and the trace diffusion‐weighted images at *b* = 1000 s/mm^2^.

	2D‐DWI	3D‐DWI		
	M0	*M*M2	M1	*M*M0
In‐plane phase encoding direction	27.8	5.6	2.7	2.8
Frequency encoding direction	28.6	4.3	4.6	3.0
Slice encoding direction	27.8	6.1	3.3	3.5
Trace diffusion‐weighted	41.7	7.7	4.9	5.4

**M0**: M_0 _= 0, **M1**: M_1 _= M_0 _= 0, **M2**: M_2_ = M_1_ = M_0_ = 0.

**TABLE 2:** SNR values before applying an apodization filter for *b* = 0 and *b* = 1000 s/mm^2^ trace diffusion‐weighted images. Results are shown for five repeated 3D‐DWI scans, along with the average, and for one 2D‐DWI scan. Measured and theoretical 3D‐to‐2D SNR ratios are also included.

	b0	b1000
SNR 2D‐DWI	37	28
SNR 3D‐DWI	39 (37, 36, 39, 42, 41)	27 *(26, 26, 28, 26, 29)*
SNR 3D/2D Measured	1.06	0.99
SNR 3D/2D Theoretical	0.98	0.86

## 5 DISCUSSION

With first and second order moment‐nulled diffusion encoding gradients, high spatial resolution and high SNR multi‐shot brain diffusion images of respectable quality can be obtained on a state of the art clinical scanner without navigator echo based phase correction. The results demonstrate that a first and second order motion‐compensated gradient scheme achieves substantial reduction, but not elimination, of motion‐related ghosting and signal loss artifacts compared to the other motion‐compensated configurations. This is in agreement with the study by Michael et al^20^ that used a high performance gradient insert for motion‐compensated multi‐shot 2D‐DWI of the brain. They reported that with first and second order moment nulling at 200 mT/m gradient amplitude shot‐to‐shot phase variations were of notable low spatial order and significantly reduced to about 10°. As expected, conventional gradient encoding is invariably associated with severe artifacts. Meanwhile, using only first order moment nulling achieves less artifact reduction than first and second order moment nulling. Some inconsistency of these observations can be attributed to subject variability and to the random timing between motion events and phase encoding shots. The low effectiveness in artifact reduction for first order moment nulling is likely due to acceleration and the elevated third order gradient moment that sensitizes for jerk (see Figure 2). Although the slice select gradient of a thick slab sequence exhibits lower amplitude, associated cross‐terms do not play a role, since the gradient is rewound before diffusion encoding starts.

An assessment of SNR gain for in‐vivo data can be difficult because tissue relaxation values reported in literature are inconsistent.^25^, ^28^ With a 92‐shot acquisition and a white matter T_1_ relaxation time of 1100 ms,^25^ the SNR advantage of a spin‐echo 3D scan over a 2D scan^2^ theoretically amounts to 2.8 for a shot‐to‐shot interval of 500 ms and to 5.3 for a shot‐to‐shot interval of 1000 ms. Given these shot‐to‐shot intervals, the 2D scan can be performed with magnitude signal averaging at no cost in overall scan time, but without the benefit of lowering Rician signal bias. With the attained shot‐to‐shot interval of 125 ms for the 2D scan, the 3D SNR advantage for an equal scan time scenario reduces to 1.4 for a 3D shot‐to‐shot interval of 500 ms (4 averages) and 1.9 for a 3D shot‐to‐shot interval of 1000 ms (8 averages). Furthermore, consideration of echo‐time related signal loss almost eliminates the 3D SNR advantage: assuming a white matter transverse relaxation time of 75 ms,^26^ the echo time penalty, that is, 119 ms TE for 3D‐DWI with $M_{0}=M_{1}=M_{2}=0$ versus 82 ms for 2D‐DWI, reduces the 3D SNR advantage to 0.86 for a 3D shot‐to‐shot interval of 500 ms and 1.14 for a 3D shot‐to‐shot interval of 1000 ms. Importantly, with increasing number of slices the 3D versus 2D SNR ratio grows according to the square root of the slice number proportion. Measured 3D versus 2D SNR ratio aligned closely with these theoretical predictions (see Table. 2) underpinning the practicality of the 3D‐DWI approach. It should be noted that estimation of noise could have been done in two ways, either by calculating the mean or the standard deviation within a ROI in the noise maps. Circumstances, such as application of an apodization filter during the reconstruction process, will alter the estimated noise and hence give different SNR values depending on whether the mean or the standard deviation is used.^29^ This will, however, not affect the 3D versus 2D SNR ratio.

The observed 3D versus 2D SNR ratio of around one does not provide a true SNR benefit for the 3D approach. Raising the number of slices would permit a more substantial advantage.

The 3D approach may thus be a practical pathway towards sub‐millimeter resolution at acceptable SNR, particularly if performed at short TR and short overall scan time. Provided the echo time penalty can be addressed with higher gradient performance, the 3D SNR advantage would also materialize at the intermediate spatial resolution evaluated in the present study.

The 3D acquisition in the present configuration, while promising, did not reliably produce images of consistent high quality. The higher signal variation that was observed with repeated scans for the 3D acquisition compared to the repeated 2D scans indicates that besides noise residual motion‐related phase shifts cause substantial signal fluctuations. Motion‐related phase shifts could be reduced by cardiac gating, which may be considered cumbersome. Alternatively, a simple navigator‐based phase correction with limited k‐space coverage, for example, 1D navigator echoes along each of the three main axes for measurement of residual phase variations could be used for simple phase‐correction.

The motion‐related phase is the product of gradient moment *M*_n_, corresponding derivatives of physiological and involuntary subject motion, and the relative weight that decreases with the factorial *n* of the Taylor expansion. While there are plenty of reports about MRI‐based velocity measurements, including measurements in brain,^6^ measurements of higher order motion derivatives are scarce. Barker et al.^19^ measured acceleration in the ascending aorta of healthy subjects with a dedicated acceleration‐sensitive MRI sequence. They observed peak accelerations between 10 and 26 m/s^2^, which is approximately the 10–20 fold numerical value of the peak systolic velocity measured in m/s.^30^ The time course plot samples presented in this publication permit an estimation of peak jerk values that scale with the same numerical value range when compared to the peak acceleration measured in m/s^2^, respectively up to 400 fold when compared to peak systolic velocity measured in m/s. Although these measures are not directly related to brain pulsatile motion, they are relevant, since it is the arterial blood flow that drives the pulsatile motion of brain tissue, i.e., the motion that is considered the dominant source of phase variations in segmented diffusion imaging.^31^ For derivatives beyond jerk, literature values for arterial blood flow could not be found, but Eager et al.^32^ reported accelerometer measurements obtained during a bounce on a gymnastic trampoline together with numerically determined higher order derivatives jerk, snap, and crackle. The numerical value observed for time measured on a seconds scale increased by approximately a magnitude with each derivative increment.

From the graphical visualization of the moments as a function of attainable gradient strength (see Figure 2) it becomes immediately apparent that at very low gradient strength, moments are very high and that higher order moments are likely to significantly contribute to the observed phase shifts in the presence of tissue motion. This explains why early diffusion imaging experiments with historically low gradient performance were frustratingly difficult, particularly when attempting a segmented acquisition. Early attempts to combat this motion sensitivity with velocity‐compensated gradients were of limited success,^16^, ^17^ since the velocity compensation resulted in a pronounced sensitivity for acceleration and higher order motion derivatives. Focusing on the upper end of the shown gradient amplitude scale, it is evident that the first order moment decreases slowly and that even with extreme gradient performance it is likely to spoil a segmented acquisition due to phase errors. Although the second order moment descends faster, it is also bound to introduce significant phase errors, irrespective of gradient performance. It is thus clear, that even with high gradient amplitudes a diffusion encoding gradient trajectory, that results in minimal phase shifts in the presence of physiological and involuntary subject motion must include first and second order moment nulling. For higher order motion, given the crude estimation of higher order motion derivatives, the rapidly diminishing weight, and the fact that for increasing gradient amplitudes gradient moments higher than *M*_2_ diminish swiftly according to a power law, one can conjecture that for very high gradient amplitudes phase shifts related to jerk may be small and phase shifts related to derivatives beyond jerk may be negligible. The graph showing the gradient lobe duration implies that at very high gradient amplitudes the resulting cost in overall gradient duration and associated echo time prolongation is modest.

As expected, ghosting artifacts observed with the motion‐compensated 3D‐DWI approach diminish as the b‐value is reduced. At *b *= 160 s/mm^2^ the artifacts reached a level that is comparable to what was observed with the single‐shot 2D multi‐slice experiments without motion compensation at *b = *1000 s/mm^2^. The *M*_3_ moment realized with 69 mT/m at *b = *64 s/mm^2^ equals the *M*_3_ moment at *b = *1000 s/mm^2^ using a maximum gradient amplitude of 300 mT/m. This supports the notion that such ultra‐strong gradients should permit motion‐compensated (*M*_0_ *= M*_1_ *= M*_2 _= 0) diffusion‐weighted imaging with segmentation at b‐values prevalent in clinical diffusion imaging applications. The gradient trajectory with velocity compensation (*M*_0_ *= M*_1 _= 0) exhibits both an *M*_2_ moment and a significantly elevated *M*_3_ moment, which implies a lower maximum b‐value with acceptable artifacts. Moreover, since the motion compensation for the second order derivative comes at virtually no cost in gradient trajectory time, using the gradient trajectory with *M*_0_ *= M*_1_ *= M*_2 _= 0 is preferred. More advanced gradient encoding waveforms allow for further echo time reduction.^33^, ^34^

Ultimately, the proposed remedies to reduce motion‐related ghosting may be used in combination. At the highest gradient strength presently available for human use,^35^ echo‐time related signal loss would be small, unless the tissue T_2_ relaxation time of interest is very short. On the other hand, at very high gradient amplitudes the contribution of concomitant field gradients^36^ cripples the gradient moment nulling in the periphery of the field‐of‐view: At 69 mT/m the moment *M*_1_ of a first and second order motion‐compensated gradient at 0.1 m off‐center equals 0.18 · 10^−3^ s^2^mT/m. While this is more than hundred times smaller than the *M*_1_ that results with the conventional uncompensated gradient configuration (see Figure 2), the concomitant field gradient does increase quadratically with gradient strength. Consequently, even with motion compensation, the resulting *M*_1_ at off‐center positions may become relevant at higher gradient amplitudes, although the shorter gradient duration that can be realized counteracts the *M*_1_ increase significantly.

With clinical multi‐slice 2D‐DWI a slice thickness below 1.5 mm is not practical. A 3D‐DWI acquisition overcomes this limitation by permitting much thinner slices suitable for multi‐planar reformatting of the image data. It may permit resolutions equivalent to what can be achieved with other MR image contrasts. This can be beneficial for the investigation of small lesions, which otherwise exhibit low contrast due to partial volume effects. Furthermore, high isotropic resolution, enabled by 3D‐DWI, would improve nerve fiber tractography, which is used for pre‐surgical planning in brain tumor patients.^37^

Higher resolution, particularly at longer scan times, may necessitate means to compensate for head motion. The addition of a second phase encoding direction, however, opens up the possibility for additional acceleration methods, such as multi‐coil acceleration or compressed sensing, which, moreover, can be applied in combination. A thick slab excitation with short repetition time introduces T_1_‐weighting. The effect of a shorter repetition time can be clearly seen in areas of cerebro‐spinal fluid (CSF) on the *b* = 0 images shown in Figure 6. Unlike images that are purely T_1_‐weighted, CSF does not appear dark since the images shown in Figure 6 are also heavily T_2_‐weighted.

The present study has limitations. Testing in only few volunteers only cannot give the full picture about clinical usefulness, since patients may be less cooperative and cause more motion‐related artifacts. The slab excitation and encoding field‐of‐view might need optimization for efficient brain imaging. Although images were acquired at two different TRs, more extensive testing is required to find the optimal TR for maximum SNR per time unit. Also, the level of acceptable T_1_‐weighting should be explored, assuming that less CSF signal in the *b* = 0 images is acceptable or even desirable to overcome truncation artefacts.^38^

## 6 CONCLUSION

In conclusion, these preliminary results indicate that the use of motion‐compensated diffusion gradients may be a viable avenue to perform single thick‐slab segmented 3D‐DWI with full brain coverage at high resolution. Unlike multi‐slab 3D‐DWI techniques, which use navigator echoes, the present approach does not rely on any second echo formation. But some basic navigation may be necessary to establish 3D‐DWI as a robust imaging alternative. The present approach clearly benefits from higher gradient strengths, since higher order gradient moments and resulting phase shifts are favorably reduced and much shorter echo times can be realized, even with moment‐nulled gradients. With such enhancements the motion compensation approach may be practical.

## CONFLICT OF INTEREST STATEMENT

The authors declare no conflicts of interest.

## References

[1] Engström M , Skare S . Diffusion‐weighted 3D multislab echo planar imaging for high signal‐to‐noise ratio efficiency and isotropic image resolution. Magn Reson Med. 2013;70(6):1507‐1514. doi:10.1002/mrm.24594 23359357

[2] Engström M , Mårtensson M , Avventi E , Skare S . On the signal‐to‐noise ratio efficiency and slab‐banding artifacts in three‐dimensional multislab diffusion‐weighted echo‐planar imaging. Magn Reson Med. 2015;73(2):718‐725. doi:10.1002/mrm.25182 24647997

[3] Chang HC , Sundman M , Petit L , et al. Human brain diffusion tensor imaging at submillimeter isotropic resolution on a 3Tesla clinical MRI scanner. Neuroimage. 2015;118:667‐675. doi:10.1016/j.neuroimage.2015.06.016 26072250 PMC4554968

[4] Holtrop JL , Sutton BP . High spatial resolution diffusion weighted imaging on clinical 3 T MRI scanners using multislab spiral acquisitions. J Med Imaging. 2016;3(2):023501. doi:10.1117/1.jmi.3.2.023501 PMC4828939 27088107

[5] Moeller S , Ramanna S , Lenglet C , et al. Self‐navigation for 3D multishot EPI with data‐reference. Magn Reson Med;84(4):1747‐1762. doi:10.1002/mrm.28231. Published online 2020. PMC7329618 32115756

[6] Maier SE , Hardy CJ , Jolesz FA . Brain and cerebrospinal fluid motion: real‐time quantification with M‐mode MR imaging. Radiology. 1994;193(2):477‐483. doi:10.1148/radiology.193.2.7972766 7972766

[7] Setsompop K , Fan Q , Stockmann J , et al. High‐resolution in vivo diffusion imaging of the human brain with generalized slice dithered enhanced resolution: simultaneous multislice (gSlider‐SMS). Magn Reson Med. 2018;79(1):141‐151. doi:10.1002/mrm.26653 28261904 PMC5585027

[8] Wu W , Koopmans PJ , Frost R , Miller KL . Reducing slab boundary artifacts in three‐dimensional multislab diffusion MRI using nonlinear inversion for slab profile encoding (NPEN). Magn Reson Med. 2016;76(4):1183‐1195. doi:10.1002/mrm.26027 26510172 PMC4854328

[9] Dai E , Wu Y , Wu W , et al. A 3D k‐space Fourier encoding and reconstruction framework for simultaneous multi‐slab acquisition. Magn Reson Med. 2019;82(3):1012‐1024. doi:10.1002/mrm.27793 31045283 PMC6831486

[10] Golay X , Jiang H , van Zijl PCM , Mori S . High‐resolution isotropic 3D diffusion tensor imaging of the human brain. Magn Reson Med. 2002;47(5):837‐843. doi:10.1002/mrm.10143 11979561

[11] Chang HC , Hui ES , Chiu PW , Liu X , Chen NK . Phase correction for three‐dimensional (3D) diffusion‐weighted interleaved EPI using 3D multiplexed sensitivity encoding and reconstruction (3D‐MUSER). Magn Reson Med. 2018;79(5):2702‐2712. doi:10.1002/mrm.26944 28940484

[12] Gao Y , Han F , Zhou Z , et al. Multishot diffusion‐prepared magnitude‐stabilized balanced steady‐state free precession sequence for distortion‐free diffusion imaging. Magn Reson Med. 2019;81(4):2374‐2384. doi:10.1002/mrm.27565 30488979

[13] Roccia E , Neji R , Benkert T , Kiefer B , Goh V , Dregely I . Distortion‐free 3D diffusion imaging of the prostate using a multishot diffusion‐prepared phase‐cycled acquisition and dictionary matching. Magn Reson Med. 2021;85(3):1441‐1454. doi:10.1002/mrm.28527 32989765

[14] Gao Y , Zhou Z , Han F , Zhong X , Yang Y , Hu P . 3D isotropic resolution diffusion‐prepared magnitude‐stabilized bSSFP imaging with high geometric fidelity at 1.5 Tesla. Med Phys. 2020;47(8):3511‐3519. doi:10.1002/mp.14195 32329081

[15] Pipe JG , Chenevert TL . A progressive gradient moment nulling design technique. Magn Reson Med. 1991;19(1):175‐179. doi:10.1002/mrm.1910190116 2046531

[16] Brockstedt S , Thomsen C , Wirestam R , et al. Use of an enhanced gradient system for diffusion MR imaging with motion‐artifact reduction. Acta Radiol. 1995;36(4‐6):662‐670. doi:10.1177/028418519503600471 8519581

[17] Clark CA , Barker GJ , Tofts PS . Improved reduction of motion artifacts in diffusion imaging using navigator echoes and velocity compensation. J Magn Reson. 2000;142(2):358‐363. doi:10.1006/jmre.1999.1955 10648154

[18] Geng R , Zhang Y , Rice J , et al. Motion‐robust, blood‐suppressed, reduced‐distortion diffusion MRI of the liver. Magn Reson Med. 2023;89(3):908‐921. doi:10.1002/mrm.29531 36404637 PMC9792444

[19] Barker AJ , Staehle F , Bock J , Jung BA , Markl M . Analysis of complex cardiovascular flow with three‐component acceleration‐encoded MRI. Magn Reson Med. 2012;67(1):50‐61. doi:10.1002/mrm.22974 21590722

[20] Michael ES , Hennel F , Pruessmann KP . Motion‐compensated diffusion encoding in multi‐shot human brain acquisitions: insights using high‐performance gradients. Magn Reson Med. 2024;92(2):556‐572. Published online 2024. doi:10.1002/mrm.30069 38441339

[21] Kwiat D , Einav S , Elad D . Possible detection of turbulent blood flow using multiparametric encoding gradients in MRI. Med Phys. 1991;18(2):316‐323. doi:10.1118/1.596678 2046622

[22] Gatenby JC , McCauley TR , Gore JC . Mechanisms of signal loss in magnetic resonance imaging of stenoses. Med Phys. 1993;20(4):1049‐1057. doi:10.1118/1.597001 8413012

[23] Wood ML , Zur Y , Neuringer LJ . Gradient moment nulling for steady‐state free precession MR imaging of cerebrospinal fluid. Med Phys. 1991;18(5):1038‐1044. doi:10.1118/1.596739 1961144

[24] Devernay F. C/C++ Minpack. Published 2007. Accessed June 2, 2022. http://devernay.github.io/cminpack

[25] Stanisz GJ , Odrobina EE , Pun J , et al. T1, T2 relaxation and magnetization transfer in tissue at 3T. Magn Reson Med. 2005;54(3):507‐512. doi:10.1002/mrm.20605 16086319

[26] Lu H , Nagae‐Poetscher LM , Golay X , Lin D , Pomper M , Van Zijl PCM . Routine clinical brain MRI sequences for use at 3.0 Tesla. J Magn Reson Imaging. 2005;22(1):13‐22. doi:10.1002/jmri.20356 15971174

[27] Uecker M , Ong F , Tamir JI , et al. Berkeley advanced reconstruction toolbox. Proc Intl Soc Mag Reson Med 2015;23:2486. Vol 23. 2015.

[28] Wansapura JP , Holland SK , Dunn RS , Ball WS . NMR relaxation times in the human brain at 3.0 Tesla. J Magn Reson Imaging. 1999;9(4):531‐538. doi:10.1002/(SICI)1522-2586(199904)9:4<531::AID-JMRI4>3.0.CO;2-L 10232510

[29] Dietrich O , Raya JG , Reeder SB , Reiser MF , Schoenberg SO . Measurement of signal‐to‐noise ratios in MR images: influence of multichannel coils, parallel imaging, and reconstruction filters. J Magn Reson Imaging. 2007;26(2):375‐385. doi:10.1002/jmri.20969 17622966

[30] Garcia J , van der Palen RLF , Bollache E , et al. Distribution of blood flow velocity in the normal aorta: effect of age and gender. J Magn Reson Imaging. 2018;47(2):487‐498. doi:10.1002/jmri.25773 28556277 PMC5702593

[31] Jiang H , Golay X , Van Zijl PCM , Mori S . Origin and minimization of residual motion‐related artifacts in navigator‐corrected segmented diffusion‐weighted EPI of the human brain. Magn Reson Med. 2002;47(4):818‐822. doi:10.1002/mrm.10102 11948746

[32] Eager D , Pendrill AM , Reistad N . Beyond velocity and acceleration: jerk, snap and higher derivatives. Eur J Phys. 2016;37(6):1‐11. doi:10.1088/0143-0807/37/6/065008

[33] Peña‐Nogales Ó , Zhang Y , Wang X , et al. Optimized diffusion‐weighting gradient waveform design (ODGD) formulation for motion compensation and concomitant gradient nulling. Magn Reson Med. 2019;81(2):989‐1003. doi:10.1002/mrm.27462 30394568 PMC6289642

[34] Aliotta E , Wu HH , Ennis DB . Convex optimized diffusion encoding (CODE) gradient waveforms for minimum echo time and bulk motion–compensated diffusion‐weighted MRI. Magn Reson Med. 2017;77(2):717‐729. doi:10.1002/mrm.26166 26900872

[35] Setsompop K , Kimmlingen R , Eberlein E , et al. Pushing the limits of in vivo diffusion MRI for the human connectome project. Neuroimage. 2013;80:220‐233. doi:10.1016/j.neuroimage.2013.05.078 23707579 PMC3725309

[36] Bernstein MA , Zhou XJ , Polzin JA , et al. Concomitant gradient terms in phase contrast MR: analysis and correction. Magn Reson Med. 1998;39(2):300‐308. doi:10.1002/mrm.1910390218 9469714

[37] Mandelli ML , Berger MS , Bucci M , Berman JI , Amirbekian B , Henry RG . Quantifying accuracy and precision of diffusion MR tractography of the corticospinal tract in brain tumors: clinical article. J Neurosurg. 2014;121(2):349‐358. doi:10.3171/2014.4.JNS131160 24905560

[38] Perrone D , Aelterman J , Pižurica A , Jeurissen B , Philips W , Leemans A . The effect of Gibbs ringing artifacts on measures derived from diffusion MRI. Neuroimage. 2015;120:441‐455. doi:10.1016/j.neuroimage.2015.06.068 26142273

